# Transcript Dynamics in Wounded and Inoculated Scots Pine

**DOI:** 10.3390/ijms22041505

**Published:** 2021-02-03

**Authors:** Vilnis Šķipars, Dainis Ruņģis

**Affiliations:** Genetic Resource Centre, Latvian State Forest Research Institute “Silava”, 111 Rigas st., LV-2169 Salaspils, Latvia; dainis.rungis@silava.lv

**Keywords:** *Pinus sylvestris*, transcriptome, *Heterobasidion annosum*, plant–pathogen interaction

## Abstract

Comparative transcriptome analysis provides a useful tool for the exploration of plant–pathogen interaction by allowing in-depth comparison of gene expression between unaffected, inoculated and wounded organisms. Here we present the results of comparative transcriptome analysis in genetically identical one-year-old Scots pine ramets after wounding and inoculation with *Heterobasidion annosum*. We identified 230 genes that were more than 2-fold upregulated in inoculated samples (compared to controls) and 116 downregulated genes. Comparison of inoculated samp les with wounded samples identified 32 differentially expressed genes (30 were upregulated after inoculation). Several of the genes upregulated after inoculation are involved in protection from oxidative stress, while genes involved in photosynthesis, water transport and drought stress tolerance were downregulated. An NRT3 family protein was the most upregulated transcript in response to both inoculation and wounding, while a U-box domain-containing protein gene was the most upregulated gene comparing inoculation to wounding. The observed transcriptome dynamics suggest involvement of auxin, ethylene, jasmonate, gibberellin and reactive oxygen species pathways and cell wall modification regulation in response to *H. annosum* infection. The results are compared to methyl jasmonate induced transcriptome dynamics.

## 1. Introduction

Scots pine (*Pinus sylvestris* L.) is a forest tree species of significant ecological and economic significance in Northern Europe. Among the most destructive diseases affecting Scots pine is root rot caused by *Heterobasidion annosum* [1]. It is essential to understand the plant–pathogen interaction to enable the development of solutions for controlling the adverse effects of the pathogen. One of the most effective ways of investigating plant responses to stress conditions is transcriptome analysis. Studies on molecular genetic aspects of conifer resistance against *Heterobasidion* sp. began before massively parallel sequencing techniques became widely available [2]. Massive parallel transcriptome sequencing analysis has the potential to deepen the pool of available information about differentially regulated genes and the likely biological effects of these changes. Some interesting data have been published identifying differentially expressed genes (DEGs) in white pine blister rust (caused by the biotrophic pathogen *Cronartium ribicola*) resistant genotypes of *Pinus monticola* [3]. Candidate genes for increased resistance of *Pinus radiata* against *Fusarium circinatum* were detected using RNA-seq [4] and transcriptome-based research on the expression of pathogenesis-related genes of *Pinus tecunumanii* after inoculation with *F. circinatum* has been published recently [5]. Transcriptome analysis can be applied more widely and provide more information compared to microarray hybridization or qPCR as it, per se, does not require detailed information about the genome of the studied organism to quantitate the transcripts of genes. Previous studies on *Heterobasidion*—conifer interaction at a transcriptome level were performed using hybridization arrays [6] in Scots pine and massively parallel sequencing in a study investigating differences in gene expression of Norway spruce genotypes with different susceptibility to *Heterobasidion* spp. infection [7]. A study describing the differences in transcriptional responses associated with virulence and defense in the interaction between *H. annosum* and *Picea abies* identified several differentially expressed genes that are likely involved in disease responses [8]. Therefore, transcriptome analysis of *P. sylvestris* responses to *H. annosum* infection will provide new information about the interaction between *P. sylvestris* and *H. annosum*. Another strategy for discovering molecular genetic information about resistance to pathogens in conifers is the identification of quantitative trait loci (QTL) [9]. The information about single nucleotide polymorphisms (SNPs) in QTLs can also be found in transcriptome data if the QTL is transcribed. Additionally, protein analysis can be used for studies of differences in stress responses [10,11]. Researchers are also studying constitutive resistance [12] and induced resistance [13]. Transcriptome studies can be focused on phytohormone-linked genes and integrated with phytohormone profiling to reveal a combined phytohormone-focused view of plant–pathogen interactions [14]. Alternatively, the impact of phytohormones on the transcriptome can be studied [15], gaining valuable information that can be used for comparisons with other treatments, as done in this study. However, to enable a thorough interpretation of transcriptome sequencing data, a reference genome or transcriptome with detailed gene annotation information is required. In comparison to other model and crop species, conifer genome resources are less comprehensive, but several genome assemblies [16,17] and transcriptomes [18,19,20] are available, as well as *H. annosum* transcriptomic and genomic resources [21,22]. The constantly growing amount of information about conifer genes and proteins deposited in public databases also means that the data obtained in experiments investigating transcriptional responses of conifers to pathogens, especially if obtained with high throughput sequencing technologies, should be periodically reexamined.

Scots pine is the dominant species in Latvia, and the breeding program produces improved germplasm for forest renewal. However, currently, selection criteria are focused on growth and stem quality characteristics. The significance of this study lies in the high economic importance of Scots pine–*H. annosum* pathosystem. Our results indicate potential candidate genes for further research, with the ultimate aim of identifying Scots pine germplasm with increased resistance to *H. annosum* infection. The specific aim of this study was to identify the changes in the transcriptome of Scots pine in response to inoculation with *H. annosum* and to clarify which of these changes are inoculation-specific. As phytohormones are important regulators of plant defense responses, the analysis and discussion were also focused on this aspect.

## 2. Results

The transcriptome sequencing resulted in ~59.1 million reads with an average length of 78 base pairs (bp). Details regarding reading count per library and mean read length are provided in Table 1.

Libraries obtained from control, wounded, and inoculated samples were mapped against an *H. annosum* reference transcriptome to confirm inoculation and to identify pathogen genes. The reads from control libraries produced at least one hit with 9190 of 13,405 *H. annosum* reference transcripts (~68.56%); for the wounded sample and inoculated sample libraries, this number is, respectively, 9225 and 11,176 (~68.82% and 83.37%). Filtering for false discovery rate-adjusted P values identified 54 transcripts “differentially expressed” between control and inoculated samples, 52 of them were “upregulated”. One “downregulated” transcript was identified comparing wounded and control samples. Supplementary Table S1 contains two sheets showing the “differential expression analysis” results for inoculated and wounded samples compared to controls. These results confirm the presence of active *H. annosum* in the inoculated samples.

The obtained number of reads per library is sufficient for meaningful RNA seq based transcript quantification and differential expression studies [23,24]. After exclusion of the outlier library, up- and downregulated transcripts were identified (Table 2).

**Figure 1:**
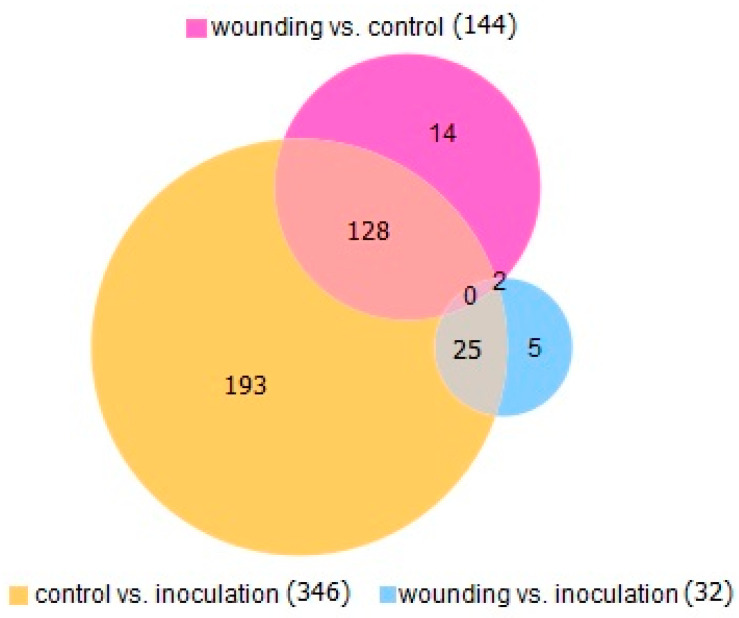
Number of DEGs depending on treatment.

The number of unique and common DEGs between different treatments is shown in Figure 1. All DEGs that overlap between treatments are differentially expressed in the same direction.

After inoculation, a transcript representing a Casparian strip membrane protein (CASP) family member was the most upregulated. Other highly upregulated transcripts included an NRT3 family protein, two Kunitz type trypsin inhibitor/alpha-amylase/subtilisin inhibitor genes, a polyadenylate-binding protein and others. The ten most upregulated transcripts after inoculation are shown in Table 3.

A transcript encoding an expansin-like protein was the most downregulated transcript (log_2_FC = 9.21). As reviewed by Cosgrove [25], expansins are involved in plant cell wall growth and stress responses (abiotic and biotic). The ten most downregulated transcripts (Table 4) include genes involved in cell wall maintenance, defense against pathogens, mitochondrial electron transport chain, water transport and water stress tolerance, cell signaling, gene expression regulation and insect resistance.

Four of the ten most upregulated wounding-induced transcripts (Table 5) are also among the ten most upregulated transcripts after inoculation. All genes in Table 5 are also upregulated in response to inoculation.

Other genes highly upregulated after wounding include peroxidases, glucan endo-1,3-alpha-glucosidases and enzymes for cysteine and phenolic compound metabolism.

The ten most downregulated genes after wounding are listed in Table 6. Two of the downregulated transcripts from this list (dehydrin and expansin-like protein) have closely related transcripts that are among the most downregulated after inoculation.

Other downregulated genes include catalases, a gene encoding an auxin-repressed protein, abscisic acid and water-stress-induced protein, chloroplast-related genes, and an anthocyanidin reductase.

The ten most upregulated annotated genes, comparing inoculation to wounding, as well as the only downregulated genes in this comparison, are shown in Table 7. Four of these genes are also among the most upregulated after inoculation (Table 3). These are the genes for the U-box domain-containing protein, CASP protein, polyadenylate-binding protein and UDP-glycosyltransferase. Additionally, an uncharacterized transcript, which, according to a BLASTn search, could represent a mitochondrial transcript, is also upregulated when comparing inoculation to wounding or control conditions. All differentially expressed genes and expression levels are presented in Supplementary Table S2.

Gene ontology (GO) data were obtained and GO term enrichment was assessed by use of Blast2GO plugin for CLC Genomics Workbench. In many cases, the transcripts produced GO descriptions of different levels of detail, which hinders apprehensible data representation. A graph representing biological processes GO classifications represented by all DEGs is provided in Supplementary Figure S1.

## 3. Discussion

### 3.1. Comparison of Differentially Expressed Genes Between Inoculated and Wounded Samples

Thirty genes were differentially expressed between wounded and inoculated samples. Such genes should be important in biotic stress management thus are potential candidate genes for further research in plant defense as polymorphisms in these genes, or their regulatory regions might favorably influence biotic stress responses. GO annotations provide information indicating the role of these DEGs in transcription, mRNA processing and translation (Table 8).

GO information about cellular localization of the proteins encoded by these DEGs shows their involvement in ribosome biogenesis, spliceosomal complex, ER membrane and plasmatic membrane (Supplementary Figure S2). Several of them are involved in signaling, cell cycle regulation, cell wall maintenance and protein homeostasis. A summary of the functions of these genes is provided in Supplementary Table S3.

### 3.2. Inoculation-Specific Changes in Gene Expression Compared to Control

**Figure 2:**
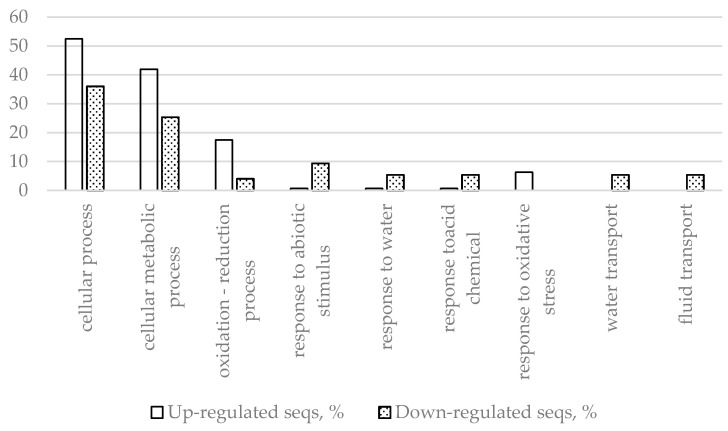
Biological processes with most up- and downregulated differentially expressed genes (DEGs) (inoculation vs. control) determined by Fisher’s exact test using *p* < 0.05 as the threshold.

**Figure 3:**
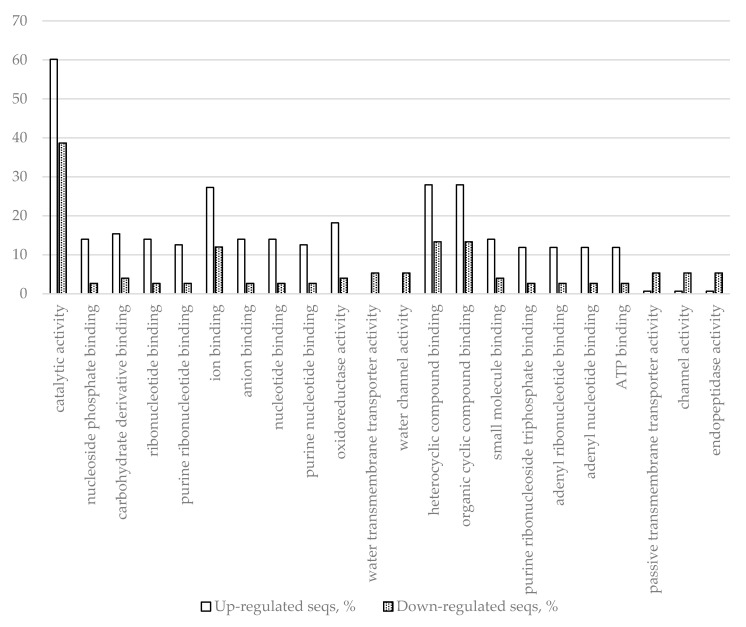
Molecular functions with most up- and downregulated DEGs (inoculation vs. control) determined by Fisher’s exact test using *p* < 0.05 as the threshold.

Another group of genes, those differentially expressed in response to inoculation, but not wounding, is intrinsically interesting for plant defense studies as genes in this group represent an inoculation-specific defense response and structural variation, and differential expression of these genes might be of interest for further research of plant resistance mechanisms. In this group are 218 genes (143 upregulated and 75 downregulated genes). Table 3 and Table 4 show information about the most up- and downregulated genes, respectively, but these tables include the section of the differential transcriptome, which overlaps with the wounding treatment. Fisher’s exact test (performed in the Blast2GO plugin for CLC genomic workbench using *p* < 0.05 as the threshold) analysis of GO frequencies revealed the most prominent differences in biological processes (Figure 2) and molecular functions (Figure 3) that the DEGs of this segment of the differential transcriptome are involved in.

In addition, to GO analysis (which often identifies different levels of detail for different DEGs, and thus can be inconvenient for result representation and interpretation), BLASTx result description analysis, simplifying the assigned gene descriptions and calculating their frequencies, can be useful (Table 9).

Four downregulated DEG descriptions include plant hormones (abscisic acid twice, auxin and gibberellin—once each). Upregulated DEG descriptions included auxin, indole-3-acetate O-methyltransferase (an enzyme converting IAA (auxin) to methyl-IAA with different biological activity [26]) (twice), 1-aminocyclopropane-1-carboxylate oxidase 5 (an enzyme involved in ethylene synthesis [27]), 12-oxophytodienoate reductase 11 (involved in methyl jasmonate (MeJa) synthesis and other signaling pathways [28]), and DMR6-like oxygenase 1, alternative name salicylate 3-hydroxylase DLO1 (converts salicylic acid (SA) to 2,3-dihydroxybenzoic acid thus negatively regulates SA content [29]). In addition to plant hormones, upregulated DEG descriptions also include glutamate synthase, which synthesizes glutamate (which, as reviewed by Qiu et al. [30], is involved in plant stress response signaling) and genes involved in Ca^2+^ (role as secondary messenger) homeostasis (calcium-binding protein, calcium-transporting ATPase 1).

The results presented in Figure 2 and Figure 3 and Table 9 show that inoculation-specific changes upregulate metabolic processes, oxidation–reduction processes, response to oxidative stress, and downregulate water transport. Both up- and downregulation of several receptor genes (receptor-like protein kinases) were detected, as well as upregulation of several genes encoding WRKY transcription factors and other genes relevant for translation. Aquaporin and dehydrin genes were downregulated, indicating that dehydration of the affected region could be a host defense strategy.

### 3.3. Genes Induced by Inoculation and Wounding

**Figure 4:**
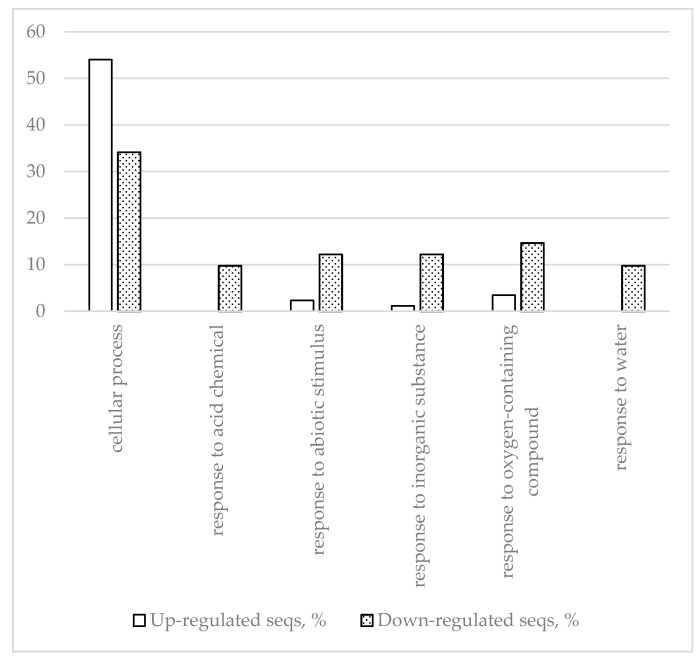
Biological processes with most up- and downregulated DEGs (inoculation/wounding vs. control) determined by Fisher’s exact test using *p* < 0.05 as the threshold.

**Figure 5:**
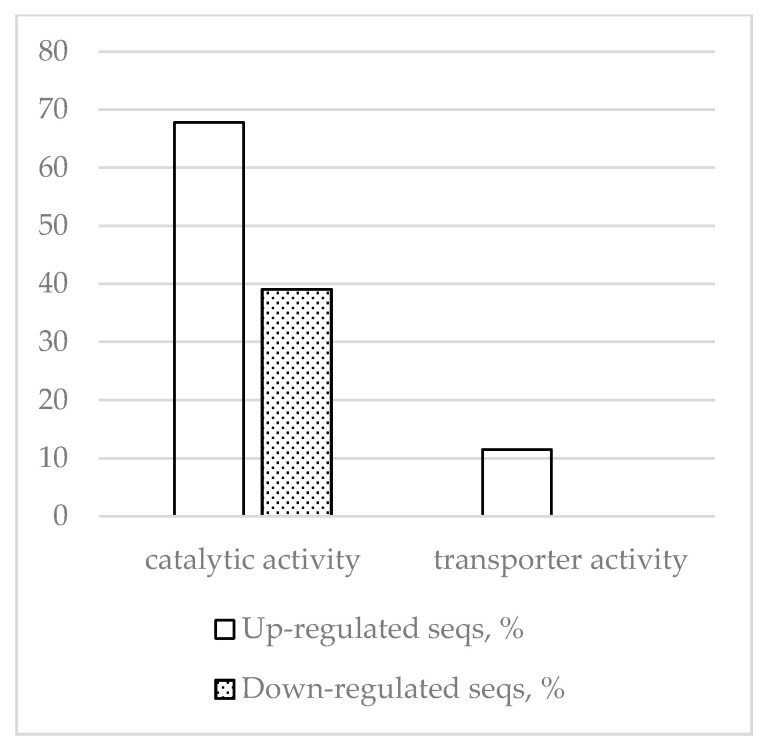
Molecular functions with most up- and downregulated DEGs (inoculation/wounding vs. control) determined by Fisher’s exact test using *p* < 0.05 as the threshold.

A total of 128 coregulated genes (87 upregulated and 41 downregulated) were detected comparing inoculation and wounding responses vs. control. Fisher’s exact test analysis of GO frequencies identified the most prominent differences between the up- and down-regulated segments of this part of the differential transcriptome related to biological processes and molecular functions (Figure 4 and Figure 5).

Table 10 provides information about the most common DEG descriptions.

The results indicate that in response to both inoculation and wounding, glucosidase genes and β- fructofuranosidase genes are upregulated, but dehydrin genes and protease/peptidase genes are downregulated. Figure 5 shows that, in contrast to inoculation-specific responses, the universal response to wounding and inoculation intensifies transporter activity. This concurs with data from Table 10 showing descriptions of “ammonium transporter” and “lysine histidine transporter” in the upregulated DEGs description list. Figure 4 shows that, compared to the inoculation-specific response, the universal response is more focused on downregulation not of genes involved in water transport, but of genes involved in “response to water,” which concurs with the “dehydrin” and “water-stress-inducible” descriptions on the downregulated DEG description list. Comparison of Table 9 and Table 10 shows that WRKY transcription factors and receptors are absent from the list of upregulated genes in Table 10.

Four upregulated DEG descriptions mention plant hormones (auxin twice, gibberellin and ethylene—once each). In addition, enzymes involved in the biosynthesis of MeJa (12-oxophytodienoate reductase and 4-coumarate—CoA ligase-like protein 7 [31]) are present in the upregulated list. Bifunctional L-3-cyanoalanine synthase/cysteine synthase, which synthesizes cysteine [32], a molecule with considerable importance in signaling and plant defense [33], is also upregulated. Hence, it is the TIFY 10B protein, which is a repressor of jasmonate responses [34]. The upregulation of genes involved in the biosynthesis of MeJa and genes for proteins repressing jasmonate responses suggests tight control of the MeJa signaling pathway.

Downregulated DEG descriptions mention auxin three times and ABA twice. One of the downregulated genes (glutamate decarboxylase gene) is responsible for the production of γ-aminobutyric acid (GABA) [35], which influences plant defense reactions in several ways [36].

Some gene descriptions occur in both up- and downregulated gene lists. Such examples include peroxidases, which, besides their role in the removal of hydrogen peroxide, are involved in the catabolism of auxin [37] and phenylalanine ammonia-lyase (PAL), which is not only involved in lignin production but also, to some degree, in the production of salicylic acid precursors [38].

### 3.4. Wounding-Specific Responses

Fourteen transcripts were differentially expressed specifically after wounding. Eight of these are upregulated, and six are downregulated compared to controls. Gene annotation descriptions of the upregulated transcripts (provided in order of decreasing induction) include a protein called enhanced disease resistance 2, a ubiquitin domain-containing protein, a nucleotide-diphospho-sugar transferase, an H-type thioredoxin, a receptor (serine/threonine-protein kinase), a cysteine-rich repeat secretory protein, a NAC domain-containing protein, one transcript was unannotated. Downregulated transcripts included (in order of increasing downregulation): ubiquitin domain-containing protein, dehydrin 7, glyceraldehyde-3-phosphate dehydrogenase, clathrin assembly protein, cytochrome P450 monooxygenase, NADH dehydrogenase subunit 5. The suppression of gene expression was more pronounced than induction—the absolute fold change for the most suppressed gene, NADH dehydrogenase subunit 5, was -55, while the most-induced gene, enhanced disease resistance 2, showed a fold change of 8.6. This short list shows that there are two differently regulated members of ubiquitin domain-containing protein in this segment of the differential transcriptome. The downregulated genes are related to water stress tolerance and general cell functions, while the upregulated genes can be linked to defense responses.

### 3.5. Transcription Factors and Signaling Pathways

#### 3.5.1. Transcription Factors

Several WRKY TFs are upregulated in response to inoculation, and no WRKY TF transcripts were downregulated. The upregulated WRKY TFs have been documented to be involved in plant defense responses. Besides the WRKY TFs, a VQ motif-containing protein transcript is induced. This is reported to interact with WRKY TFs, possibly influencing resistance. Other upregulated TFs include an ET-responsive TF (transcription activator), a PAR1 protein gene (involved in growth regulation) and others.

The downregulated TF includes zinc finger CCCH domain-containing protein 20 (reported to respond to oxidative stress), a NAC domain-containing protein (involved in auto-degradative processes in sieve element formation) and high mobility group B protein 1 (involved in stress tolerance and plant growth). A considerable proportion of differentially regulated TFs are inoculation specific (not differentially regulated in response to wounding).

#### 3.5.2. E3 Protein Ligase Complex

A notable difference between control and inoculated samples is the upregulation of several E3 protein ligase complex transcripts; no such transcripts are downregulated. E3 protein ligases ubiquitinate proteins, thus marking them for degradation. At the same time, several genes encoding proteins involved in the regulation of transcription and translation are upregulated after inoculation, suggesting a targeted attempt by the host organism to achieve homeostasis of specific proteins or to degrade some proteins to provide building blocks for the synthesis of proteins involved in defense mechanisms. Some of these genes identified in this study represent an inoculation-specific response.

#### 3.5.3. Auxin

Several up- and downregulated genes indicate regulation of auxin (IAA) levels. An auxin transporter for auxin influx is upregulated (inoculation-specifically) in addition to transcripts encoding indole-3-acetate O-methyltransferases, which convert IAA to biologically inactive IAA methyl ester (MeIAA). A methylesterase 17 analog involved in the reverse process–synthesis of IAA from MeIAA is inoculation-specifically downregulated. Several other genes involved in auxin metabolism and homeostasis regulation as well as in the regulation of auxin-responsive gene expression are differentially expressed, suggesting a change in intracellular auxin level as a response to inoculation, part of the response being inoculation-specific. The mentioned methylesterase is also involved in the metabolism of jasmonic acid (JA) and SA.

#### 3.5.4. Jasmonic Acid

Expression of genes influencing jasmonic acid metabolism and the regulation of jasmonate-responsive genes is mostly similar for inoculation and wounding treatments. Transcription of TIFY 9 and TIFY 10 genes is upregulated. They are reported to repress jasmonate responses. An IAA-amino acid hydrolase, involved in JA metabolism, is also upregulated, as are two 12-oxophytodienoate reductases, also involved in biosynthesis of JA. A JA responsive leucoanthocyanidin synthase is downregulated. Overall, the number of DEGs related/responsive to JA are lower than for auxin and ABA.

#### 3.5.5. Gibberellin

Similar to JA regulation, gene expression of gibberellin metabolism genes and gibberellin responsive genes do not show a strong inoculation specific pattern. The gibberellin 2-β-dioxygenase gene transcription is induced after inoculation. This enzyme oxidizes active gibberellin into an inactive form and is involved in homeostasis of gibberellin. Oxoglutarate/iron-dependent dioxygenase, which, according to the InterPro database, could be involved in synthesis of gibberellin, is slightly upregulated. Transcription of a gibberellin regulated protein is downregulated. These data could indicate a decrease of gibberellin influence after wounding/inoculation.

#### 3.5.6. GABA

Synthesis of GABA may be inhibited, as the glutamate decarboxylase gene is downregulated in response to inoculation and wounding. A recent review of GABA signaling is provided by Fromm [39]. GABA metabolism is linked with ROS levels [40], and glutamate decarboxylase is regulated by calmodulin [41]. Another calmodulin-binding protein, the transcription of the respective gene of which is slightly upregulated after inoculation (but not wounding), is a calcium-transporting ATPase 1. As calmodulin interacts with Ca^2+^ [42], this establishes a link between GABA signaling and Ca^2+^ signaling. One of the inoculation-specifically upregulated genes encodes a probable calcium-binding protein (annotated as CML-13, member of calmodulins), which would make a stronger case for GABA-calcium signaling interaction, yet this annotation needs to be confirmed due to a lack of detailed information about this protein.

#### 3.5.7. ABA

A larger number of ABA-responsive/signaling related genes are differentially regulated, some of them in an inoculation-specific manner. Ten genes are upregulated (two more than 4-fold), and six are downregulated (five more than 4-fold). This is more than for any other single phytohormone related genes (multiphytohormone-responsive genes excluded). The most upregulated transcript (not inoculation specific) is most similar to chloroplastic magnesium–chelatase subunit, a positive regulator of ABA signaling. The rest of the upregulated genes involving ABA are described to be ABA-responsive. The exception is a transcript for a U-box containing protein, which possibly downregulates ABA biosynthesis.

Except for a PR10 protein, the downregulated ABA-responsive protein genes represent either ABA and water stress-induced proteins or embryo-abundant proteins, response to water stress (or involvement in damage prevention from water stress) being a common feature in addition to ABA-responsiveness. As transcription of aquaporin genes is specifically downregulated after inoculation, the downregulation of these genes may be a consequence of this. The downregulation of dehydrins (mixed inoculation-specificity of downregulation), which also protects from water stress-induced damage, is clearly pronounced in the inoculated samples.

#### 3.5.8. Ethylene and Salicylic Acid

Expression of a gene for 1-aminocyclopropane-1-carboxylate oxidase, which is involved in ethylene biosynthesis, is slightly induced specifically in response to inoculation. A gene encoding a DMR6-like oxygenase, which converts SA to 2,3-dihydroxy benzoic acid, is upregulated in response to inoculation suggesting downregulation of SA signaling.

#### 3.5.9. Genes Associated with Multiple Phytohormones

A number of genes involved in the metabolism of, or response to, more than one phytohormone are differentially regulated. Only the methylesterase mentioned in context with auxin metabolism shows an expression fold change (absolute) exceeding four. The HSPRO2-like protein gene is suggested to be downregulated in response to JA and ET [43]; however, in this study, it was upregulated. However, strictosidine synthase is reported to be downregulated by auxin [44] and induced by jasmonate [45], and it was upregulated in this study. This may be the effect of fungal elicitors [44].

Two of the slightly suppressed genes in this group are functionally linked. These are lipase-like PAD4 and EDS1L-like protein genes. PAD4 probably leads to SA accumulation and, together with EDS1, seems to repress the ET/JA defense pathway. EDS1L and PAD4 are especially important in early defense responses [46,47]. The other downregulated genes are a chalcone synthase (responsive to auxin and JA), phospholipase D alpha (involved in wound induction of JA- and ABA-induced stomatal closure) and phenylalanine ammonia-lyase (in addition to lignin biosynthesis, is also involved in SA catabolism).

The expression of the PAD4 and EDS1L genes, as well as a chalcone synthase gene and one of the transcripts representing phospholipase D alpha, are suppressed while HSPRO2 is induced in an inoculation-specific manner.

#### 3.5.10. Calcium

Ca^2+^ ions are important in the regulation of cellular processes. A gene encoding a probable Ca-binding protein, CML13, is upregulated. A glutamate receptor and Ca-transporting ATPase, both involved in Ca homeostasis, are also upregulated. All three genes are upregulated in an inoculation-specific manner.

#### 3.5.11. Water Transport and Drought Stress

Water transport proteins (mostly aquaporins) are downregulated in response to inoculation. Drought damage prevention/water stress-responsive proteins are downregulated either specifically in response to inoculation or also downregulated in response to wounding, thus serving as another example of distinct regulation patterns for different genes from the dehydrin family. Most of the dehydrins and water stress-induced proteins are downregulated in response to wounding as well, but the most downregulated dehydrin and ABA-water-stress-induced protein gene analogs are the ones that represent an inoculation specific response.

#### 3.5.12. Reactive Oxygen Species Balance

Expression of 25 oxidative stress and ROS homeostasis-related genes are induced in response to inoculation, 17 of them with abs. FC > 4 while ten such genes are downregulated, of which six with abs. FC > 4. This is an indication of the significant role of ROS homeostasis in host defense responses in this study. Involvement of ROS in plant defense both directly and indirectly through signaling cascades and involvement in cell wall maintenance is well-established [48]. Eleven upregulated genes and only three downregulated genes represent an inoculation-specific response. Furthermore, the three most upregulated genes showed inoculation-specific regulation.

#### 3.5.13. Proteases and Proteinase Inhibitors

More proteases are downregulated than upregulated (nine vs. six), and more proteinase inhibitors are upregulated than downregulated (four vs. one). Proteinase inhibitors could represent a wounding-related defense response against herbivores. The up- and downregulated proteinases/peptidases represent the same groups of enzymes, probably representing a more detailed regulation of specific protein levels or tissue-specific regulation, which were not addressed in this study. The proteinase inhibitors are not regulated in an inoculation-specific manner, while some proteinase/peptidase genes show inoculation-specific regulation, mostly suppression.

#### 3.5.14. Other Genes

Genes coding proteins involved in detoxification, signaling, photosynthesis, synthesis of organic compounds, including compounds with antifungal activity, lignin biosynthesis, nitrate assimilation, transport of sugars and proteins directly involved in defense against fungal pathogens (chitinase, glucan endo 1,3-α glucosidases) and others are upregulated. Stilbene synthase genes and glucan endo 1,3-α glucosidase genes and chitinase genes are upregulated after both inoculation and wounding, but a laccase (associated with lignin degradation and detoxification of lignin-derived products, a RING-H2 finger protein (associated with early steps of defense signaling), a galacturonosyltransferase protein (involved in pectin biosynthesis) and other genes show inoculation-specific upregulation.

The inoculation-specific downregulated genes include genes for proteins directly involved in antimicrobial defense (antimicrobial peptide 1 and thaumatin-like proteins (a.k.a., PR-5 proteins)). These genes are typically members of larger gene families with different temporal, spatial and pathogen-type-specific expression patterns, so this is not unexpected.

Several of the downregulated genes are involved in photosynthesis; many are annotated as genes for receptors with protein kinase activity.

Other downregulated genes are annotated as expansin-like protein, xyloglucan endotransglucosylase/hydrolase and pectin methyltransferase genes. These are involved in cell wall maintenance, permeability regulation and cell-to-cell adhesion. An expansin-like protein gene is the most suppressed gene. If the downregulation of this gene results in the decrease of cell wall water permeability, it could be functionally consistent with the downregulation of aquaporins and suppression of drought-protective proteins. Dehydration and changes in ROS levels in response to infection are common observations, and transport of H_2_O_2_ by aquaporins might indicate an additional function of these proteins in plant defenses, as discussed in a review by Afzal et al. [49]. These authors also explain the reasons for difficulties in the interpretation of these results—a differential expression of very similar genes within and between species.

### 3.6. Comparison to MeJa Treatment

Comparison of the data in this paper with the data from Kānberga-Siliņa et al. [50] (reanalyzed using CLC Genomics Workbench and Blast2GO plugin), revealed that the number of DEGs differs (Table 11) (same logFC threshold as in Kānberga-Siliņa et al. (≥|2|) is used for both NGS data sets).

Of the 119 differentially regulated genes in response to methyl jasmonate treatment, only a few were common with responses to inoculation or wounding. Expression of eight MeJa-induced and three MeJa-suppressed genes was also differentially regulated in the inoculation/wounding experiment. Comparison of regulation of these genes in response to different treatments is presented in Table 12.

The low number of overlapping DEGs between the MeJa treatment and the inoculation experiment suggests that the MeJa signaling pathway is not the major signaling pathway in defense response to inoculation with *Heterobasidion* in Scots pine, in contrast to the case in Norway spruce [51]. However, the age of plant materials utilized in this experiment differs to that of Arenrup et al. [51], as well as experimental procedures of treatments.

For a larger scale comparison of the upregulated parts of transcriptomes obtained after MeJa treatment or inoculation, simplified Blast2GO annotation frequency analysis was performed (Table 13) showing increased frequency for chalcone synthase clavaminate synthase, chitinase and benzyl alcohol O-benzoyltransferase in the annotations for the MeJa treatment. The annotations of the differential transcriptome from inoculated samples show increased frequency of peroxidases, different receptors, WRKY transcription factors and proteases/peptidases.

The comparison suggests that a signaling pathway influencing WRKY transcription factors could be significant in defense response to necrotrophic fungal infection. However, there are some similarities between inoculation and MeJa treatment responses—oxophytodienoate reductase is involved in MeJa metabolism [28], and 2-methyl-3-buten-2-ol synthase is involved in terpene metabolism [52], as is stilbene synthase. Linoleate lipoxygenase is responsive to MeJa [53], as are several WRKY transcription factors [54]. In addition, the samples analyzed in this study may represent an early stage in the methyl jasmonate pathway in the inoculated samples, in comparison to the MeJa treatment study, which directly investigated the later stage of this pathway (after MeJa treatment).

The few observed overlapping genes in the DEG list and differences in the most upregulated genes suggests a significantly more complex signaling network than a methyl jasmonate centered pathway in the reaction of Scots pine to inoculation with *H. annosum*. Based on the obtained results, water transport prevention and prevention of dehydrin-based mitigation of draught stress is a possible strategy of Scots pine defense against infection. The transcriptome analysis also shows that many genes are involved in both ROS and protein homeostasis. However, annotation of Scots pine genes is not complete, and meaningful information about the possible functions of many differentially expressed transcripts was not available, therefore the role of several transcripts involved in these defense responses needs to be further investigated. In addition, differential gene expression profiles should be investigated in a range of Scots pine germplasm, to investigate the transcriptomic differences between individuals and their relation to differential resistance to pathogens.

## 4. Materials and Methods

One-year-old *P. sylvestris* ramets obtained by grafting of twigs of the individual Ja2-III-4 were used for the experiment. They were inoculated or wounded on September 5, 2016 and collected one day after treatment. Analysis of samples collected at similar time points after inoculation have been previously reported in other Scots pine–*H. annosum* studies [6,55]. Agar containing *H. annosum* mycelia (isolate V Str 28) was used for inoculation. Before inoculation, the bark was removed with a scalpel after the removal of needles at the treatment area. The media containing the mycelia was applied, with the upper surface of media facing the wounded area. For wounded samples, sterile agar media was applied instead of media containing *H. annosum*. For control samples only the needles were removed from the same height of the sapling at which the treatment of the other samples was done. The trees were kept outside before and during the experiment. Air temperature at the day of inoculation was +17 °C and +19 °C at the day of sample collection. For sample collection, an ~3 cm long fragment of the tree stem containing the area of inoculation/wounding and ~1 cm of the surrounding area in both directions was excised and placed into a two mL test tube, which was then frozen in liquid nitrogen and stored at −80 °C until RNA extraction.

RNA was extracted from a cross-section of the area of the stem where the manipulations had been performed. The RNA was extracted by use of Genomic DNA purification kit (#K0512, Thermo Fisher Scientific, Vilnius, Lithuania) and a modified protocol for RNA extraction [56]. The integrity of the obtained RNA samples was assessed on the 2100 Bioanalyzer (Agilent, Santa Clara, CA, USA) using an RNA nano chip following the manufacturer’s instructions. RNA integrity (RIN) values of the samples used in downstream analysis exceeded 7.

Ribosomal RNA was removed using the RiboMinus™ Plant kit for RNA-Seq, and the transcriptome libraries were prepared using the ion total RNA-Seq Kit v2 (both kits from Thermo Fisher Scientific, Waltham, MA, USA). Further sequencing procedures, including emulsion PCR and ion torrent sequencing on the Ion Proton instrument (Thermo Fisher Scientific, Waltham, MA, USA) using the ion PI chip, were performed at the Latvian Biomedical Research and Study Center.

For the data analysis, CLC Genomic Workbench software 12.1 (Qiagen, Venlo, The Netherlands) was used. The main steps of the analysis included barcode and adapter trimming, quality trimming, short read (<15 nt) filtering, read mapping to the reference transcriptome (from Wachowiak et al. [20], containing 40,798 sequences), differential gene expression analysis and transcript annotation (using Blast2GO PRO plugin v. 1.12.11 for the CLC Genomic Workbench software (BioBam Bioinformatics, Valencia, Spain)). Quality trimming settings: quality trim enabled, quality limit 0.05, ambiguous trim enabled, ambiguous limit 2, adapter trimming—automatic, discard short reads enabled, min. no. of nucleotides per read—15, max. no. of nucleotides per read—1000. RNA-Seq reference settings: one reference sequence per transcript, spike-in control handling disabled. RNA-Seq mapping settings: mismatch cost 2, insertion cost 3, deletion cost 3, length fraction 0.8, similarity fraction 0.8, auto-detect paired distances enabled, strand specificity—both, max. no. of hits per reading–10. RNA-Seq expression settings: expression value—total counts, calculate an expression for genes without transcripts enabled. In the CLC Genomic Workbench software metadata tables are used to assign information about treatment type and repeat number to the libraries. This allows this software to take the fluctuations in gene expression among different replicates into account when calculating the fold change, FDR p and other values. Annotation was done using the eukaryotic subset of the nonredundant protein sequences database (database name “nr v5” from NCBI). Nine of the reference sequences were found by BLAST analysis to probably be contaminants (of arthropod, fungal and bacterial origin) and were removed prior to further analysis, they are highlighted in red in Supplementary Table S2. Three biological replicates were used for the inoculated samples, as recommended [24]. However, only two biological replicates of wounded samples were available as principal component analysis (using normalized log CPM (count per million) values as input) during quality control steps indicated a deviation in one of the libraries (wounded sample, library 25S). Two biological replicates were used for control samples. *H. annosum* transcriptome data [57] was used to identify the presence of *H. annosum* sequences in the control, wounded and inoculated libraries. For comparison of obtained results with transcriptome dynamics in response to methyl jasmonate treatment, the analysis settings were as reported previously [50]. For a description of DEGs in Section 3.5. of the discussion the primary resource used was the information about proteins with names matching to the Blast2GO-generated description from UniProt (https://www.uniprot.org/).

## 5. Conclusions

Our results show that there are significant differences between the effects of inoculation with *H. annosum* and wounding on the transcriptome of Scots pine. The main differences are the higher expression of TFs and genes involved in the E3 protein ligase complex, as well as changes in expression of different receptor genes in inoculated samples compared to wounded samples. ROS homeostasis regulation, water transport regulation and drought stress damage prevention mechanisms are significant at this stage of *H. annosum* infection, as expression profiles of genes involved in these processes differ between inoculated and wounded samples. Concerning phytohormones, it is evident that one day post-inoculation, there is no clear main pathway regulating plant defense against inoculation. Results show that the cellular levels of several phytohormones (auxin, jasmonic acid, gibberellin and others) are regulated in response to the treatments. The low level of similarity between the response to MeJa treatment and wounding/inoculation strongly suggests that the MeJa pathway is not the main pathway regulating plant defense in this study. Several genes encoding ABA-responsive proteins show expression differences comparing inoculation and wounding treatments and are probably involved in water transport. However, the regulation of aquaporins is varied and the extent to which ABA is important in this process in inoculated samples should be further studied. This study revealed inoculation specific responses, which can be further explored. One of the most promising area for additional research is the regulation of WRKY transcription factor genes. This initial research provides the basis for further studies, examining the diversity of responses in a range of Scots pine germplasm, identifying potential sources of resistance to *H. annosum*, which can be incorporated into the Scots pine breeding program.

## Figures and Tables

**Table 1:** Read count and read length of transcriptome sequencing libraries.

Library Name	Treatment	Reads	Mean Read Length, bp
26S	Control	8,403,116	79
27S	Control	5,338,286	73
23S	Wounding	5,679,288	90
25S *	Wounding	3,386,611	82
29S	Wounding	9,003,982	69
21S	Inoculation	9,821,725	95
30S	Inoculation	7,669,090	61
34S	Inoculation	9,815,442	78

* omitted from data analysis due to deviation principal component analysis.

**Table 2:** Number of significantly up- or downregulated transcripts depending on treatment.

Compared	Number of Upregulated Transcripts	Number of Downregulated Transcripts
Inoculated vs. control	230	116
Wounded vs. control	96	48
Inoculated vs. wounded	30	2

Absolute fold change (Abs. F. C.) ≥ |2|; false discovery rate (FDR) adjusted *p* < 0.05.

**Table 3:** Eleven (10 annotated) most upregulated transcripts after inoculation (compared to control).

Mapping Reference ID	Annotation (BLASTx)	Accession	Log₂ FC (Control vs. Inoculation)	FDR *p*-Value
comp45373_c0_seq2	---NA---	mitochondrial sequence	−9.05	2.36 × 10^−8^
comp52925_c0_seq1	CASP-like protein 5A2	P0DI70	−6.92	0
comp36432_c0_seq1	NRT3 family protein	AQX43117	−6.77	2.08 × 10^−4^
comp53900_c0_seq3	Alpha-amylase/subtilisin inhibitor-like	XP_004488980	−6.72	4.61 × 10^−8^
comp53900_c0_seq2	Kunitz-type trypsin inhibitor	OMO78033	−6.63	3.85 × 10^−11^
comp50080_c0_seq8	Polyadenylate-binding protein 2 isoform X1	RWR79297	−6.33	1.48 × 10^−6^
comp55480_c0_seq16	UDP-glycosyltransferase 86A1	XP_008779947	−5.99	4.57 × 10^−3^
comp10846_c0_seq1	U-box domain-containing protein 6-like isoform X1	RWR96867	−5.79	6.5 × 10^−10^
comp50991_c0_seq1	12-Oxophytodienoate reductase	XP_006853946	−5.71	2.93 × 10^−5^
comp38130_c0_seq1	PB1 domain-containing protein	GAV63268	−5.71	0
comp52813_c0_seq2	Phosphoenolpyruvate carboxykinase (ATP)	XP_030528286	−5.58	1.2 × 10^−2^

**Table 4:** Ten most downregulated transcripts after inoculation.

Mapping Reference ID	Annotation (BLASTx)	Accession	Log₂ FC (Control vs. Inoculation)	FDR *p*-Value
comp53647_c0_seq1	Expansin-like protein	ADM74637	9.21	9.42 × 10^−3^
comp49135_c0_seq1	Antimicrobial peptide 1	AAL05052	9.19	1.59 × 10^−2^
comp50799_c0_seq2	Cytochrome b-c1 complex subunit Rieske-4, mitochondrial-like	XP_024197491	8.16	3.48 × 10^−2^
comp41900_c0_seq2	Chaperone protein dnaJ 10	XP_006849900	5.75	1.96 × 10^−3^
comp42474_c0_seq1	Aquaporin TIP2-1	XP_028756179	5.24	9.89 × 10^−4^
comp49517_c0_seq4	Serine/threonine-protein kinase	PSR96031	5.11	2.8 × 10^−2^
comp55091_c0_seq2	Histone-lysine N-**methyltransferase** SUVR5 isoform X1	XP_020530211	5.02	3.37 × 10^−3^
comp54141_c0_seq13	Dehydrin 7 protein	CAD54624	4.82	7.77 × 10^−6^
comp54141_c0_seq10	Dehydrin 5	CAD54624	4.38	1.28 × 10^−2^
comp54908_c0_seq3	Subtilisin-like protease SBT3.5	XP_020526525	4.37	3.48 × 10^−2^

**Table 5:** Ten most upregulated transcripts after wounding.

Mapping Reference ID	Annotation (BLASTx)	Accession	Log₂ FC (Control vs. Wounding)	FDR *p*-Value
comp36432_c0_seq1	NRT3 family protein	AQX43117	6.88	4.17 × 10^−4^
comp53900_c0_seq3	Alpha-amylase/subtilisin inhibitor-like	XP_004488980	6.39	1.4 × 10^−6^
comp53900_c0_seq2	Kunitz-type trypsin inhibitor	OMO78033	5.84	1.11 × 10^−7^
comp50991_c0_seq1	12-Oxophytodienoate reductase 3	XP_006853946	5.14	1.39 × 10^−3^
comp47322_c0_seq1	4-Coumarate-CoA ligase-like 7	XP_021648414	4.72	3.8 × 10^−5^
comp49557_c0_seq4	Lipid transfer-like protein VAS	XP_021646589	4.66	7.15 × 10^−5^
comp44972_c0_seq1	Probable UDP-N-acetylglucosamine-peptide N-acetylglucosaminyltransferase SEC	XP_010926674	4.48	3.24 × 10^−2^
comp54755_c0_seq16	2-Methyl-3-buten-2-ol synthase	ADZ45514	4.47	2.27 × 10^−8^
comp50925_c0_seq1	Protein flp	PFX34647	4.38	4.45 × 10^−2^
comp39845_c0_seq1	Class VII chitinase	QEL09553	4.19	5.55 × 10^−3^

**Table 6:** Ten most downregulated transcripts after wounding.

Mapping Reference ID	Annotation (BLASTx)	Accession	Log₂ FC (Control vs. Wounding)	FDR *p*-Value
comp55025_c0_seq2	NADH dehydrogenase subunit 5	AEB54948	−5.79	2.08 × 10^−2^
comp54020_c0_seq2	Aspartyl protease ED3-like	XP_031372971	−5.77	1.13 × 10^−6^
comp54141_c0_seq7	dehydrin 5	CAD54624	−5.38	1.56 × 10^−3^
comp52309_c0_seq4	Serine/threonine protein phosphatase 2A regulatory subunit B beta-like	XP_020276947	−4.70	8.94 × 10^−3^
comp27926_c0_seq1	Nonspecific lipid-transfer protein 2-like	KZV55795	−4.08	1.76 × 10^−4^
comp53647_c0_seq2	Expansin-like protein	PSS08250	−3.83	2.56 × 10^−3^
comp53589_c0_seq1	Cytochrome P450 monooxygenase CYP736B	ACN89833	−3.69	3.18 × 10^−2^
comp55331_c0_seq2	Putative clathrin assembly protein At2g25430	XP_008784532	−3.60	9 × 10^−3^
comp53823_c0_seq2	Glyceraldehyde-3-phosphate dehydrogenase 3, cytosolic	P34924	−3.44	3.46 × 10^−3^
comp54141_c0_seq5	Dehydrin	CCG34065	−3.24	5.83 × 10^−5^

**Table 7:** Eleven most upregulated (10 annotated) and two downregulated genes after inoculation, compared to wounding.

Mapping Reference ID	Annotation (BLASTx)	Accession	Log₂ FC (Wounding vs. Inoculation)	FDR *p*-Value
comp10846_c0_seq1	U-box domain-containing protein 6-like isoform X1	RWR96867	−7.48	6.72 × 10^−5^
comp45373_c0_seq2	---NA---	Mitochondrial sequence	−7.14	0
comp52925_c0_seq1	CASP-like protein 5A2	P0DI70	−6.72	0
comp50080_c0_seq8	Polyadenylate-binding protein 2 isoform X1	RWR79297	−6.27	1.24 × 10^−5^
comp38130_c0_seq1	PB1 domain-containing protein	GAV63268	−6.18	0
comp1010735_c0_seq1	60S ribosomal protein L13a-4-like	XP_024356575	−5.02	3.18 × 10^−10^
comp40240_c0_seq2	Pre-mRNA-splicing factor ISY1 homolog	XP_006854550	−4.93	0
comp52309_c0_seq4	Serine/threonine protein phosphatase 2A regulatory subunit B beta-like	XP_020276947	−4.65	1.78 × 10^−2^
comp40935_c0_seq1	NAC domain-containing protein 86-like	XP_026666241	−4.48	5.06 × 10^−3^
comp55480_c0_seq16	UDP-glycosyltransferase 86A1	XP_008779947	−4.10	6.42 × 10^−3^
comp54163_c0_seq3	L-type lectin-domain-containing receptor kinase S.4-like	XP_010937574	−4.08	2.46 × 10^−10^
Downregulated				
comp55005_c0_seq1	Paladin isoform X2	XP_006841555	5.11	9.72 × 10^−6^
comp41900_c0_seq2	Chaperone protein dnaJ 10	XP_006849900	6.28	1.75 × 10^−3^

**Table 8:** Frequency of GO descriptions (GO term level 7) for the transcripts differentially expressed between inoculation and wounding.

Biological Process	Number of Sequences
Protein phosphorylation	2
ncRNA metabolic process	2
ncRNA processing	2
Nucleic acid-templated transcription	1
mRNA splice site selection	1
Spliceosomal conformational changes to generate catalytic conformation	1
mRNA processing	1
tRNA modification	1
rRNA processing	1
Cellular protein-containing complex assembly	1
Protein modification by small protein conjugation or removal	1
Regulation of dephosphorylation	1
Regulation of nucleic acid-templated transcription	1
Chlorophyll metabolic process	1
Regulation of phosphoprotein phosphatase activity	1
Regulation of protein dephosphorylation	1
Protein dephosphorylation	1
Spliceosomal complex assembly	1
mRNA metabolic process	1
Generation of catalytic spliceosome for second transesterification step	1
Porphyrin-containing compound biosynthetic process	1
RNA splicing	1

**Table 9:** Comparison of most frequent simplified DEG descriptions between inoculation-specific up- and downregulated transcriptome segments (total number of upregulated DEGs = 143, downregulated DEGs = 75).

Description of Upregulated DEGs	Quantity	Description of Downregulated DEGs	Quantity
Receptor	9	Receptor	5
WRKY transcription factor	5	Aquaporin	4
Peroxidase	4	Protease/proteinase	4
Protease/proteinase	3	Embryo- (late embryogenesis-) abundant protein	3
Methyltransferase	3	ABA-induced protein	2
U-box domain-containing protein	3	Cytochrome	2
UDP-glycosyltransferase	3	Dehydrin	2
Benzyl alcohol O-benzoyltransferase	2	EDS1L	2
Chitinase	2	NRT1/ PTR family protein	2
Cinnamyl-alcohol dehydrogenase	2T	thaumatin-like protein	2
Cytochrome P450	2		
E3 ubiquitin-protein ligase	2		
Glutathione peroxidase	2		
Indole-3-acetate O-methyltransferase	2		
PAR1 transcription factor	2		
Pentatricopeptide repeat-containing protein	2		
Ribosomal protein	2		
Sugar transport protein	2		
Tau class glutathione S-transferase	2		
Thioredoxin reductase	2		
tRNA modifying enzyme	2		

**Table 10:** Comparison of most frequent simplified DEG descriptions between the up- and downregulated parts of the overlapping inoculation and wounding induced response (total number of upregulated DEGs = 87, downregulated DEGs = 41).

Description of Upregulated DEGs	Quantity	Description of Downregulated DEGs	Quantity
Glucosidase	6	Dehydrin	4
Beta-fructofuranosidase	3	Protease/peptidase	4
Chitinase	3	Peroxidase/catalase	3
Peroxidase	3	Chlorophyll a/b binding protein	2
12-Oxophytodienoate reductase	2	receptor	2
Ammonium transporter	2	Water-stress-inducible	2
E3 Ubiquitin-protein ligase	2		
Linoleate lipoxygenase	2		
Lysine histidine transporter	2		
Protease/peptidase	2		
Reticuline oxidase	2		
Stilbene synthase	2		
TIFY protein	2		
UDP-glycosyltransferase	2		

**Table 11:** Comparison of transcriptional responses of two-year-old MeJa-treated Scots pine trees to and one-year-old wounded or inoculated (with *H. annosum*) Scots pine trees.

	MeJa Treatment	Wounding	Inoculation
Upregulated	98	65	164
Downregulated	21	26	61
Total	119	91	225

**Table 12:** Regulation of the overlapping DEGs of the MeJa and inoculation/wounding experiments.

Reference Transcript ID	Gene Description (Blast2GO Generated)	MeJa	I	W
comp51296_c0_seq1	Glucan endo-1,3-alpha-glucosidase agn1	+	+	+
comp54755_c0_seq16	2-Methyl-3-buten-2-ol synthase	+	+	+
comp55507_c0_seq1	peroxidase 12	+	+	NS
comp55437_c0_seq1	Alpha carbonic anhydrase 7-like	+	+	NS
comp36780_c0_seq1	protein TIFY 10B	+	NS	+
comp49135_c0_seq1	Antimicrobial peptide 1	+	-	NS
comp50836_c0_seq3	Abscisic acid and water-stress-induced protein	+	-	NS
comp53758_c0_seq2	Leucoanthocyanidin dioxygenase-like	+	-	NS
comp52946_c0_seq3	Embryo-abundant protein	-	-	NS
comp55009_c0_seq7	BURP domain protein RD22-like isoform X1	-	-	NS
comp54020_c0_seq5	Aspartyl protease ED3	-	-	NS

MeJa—treatment with methyl jasmonate, I—inoculation, W—wounding, plus sign—upregulation, minus sign—downregulation of gene expression, NS—change not statistically significant (FDR *p*-value ≥ 0.05 or logFC < |2|).

**Table 13:** Comparison of most frequent simplified Blast2GO annotations of upregulated DEGs between the inoculation and MeJa treatment experiments (total number of upregulated DEGs after inoculation = 164, upregulated DEGs after MeJa treatment = 98).

Inoculation		MeJa Treatment	
Simplified Description	Count	Simplified Description	Count
Peroxidase	7	Chalcone synthase	4
Receptor	7	Clavaminate synthase	4
WRKY transcription factor	6	Benzyl alcohol O-benzoyltransferase	3
Protease/peptidase	5	chitinase	3
UDP-glycosyltransferase	5	2-Methyl-3-buten-2-ol synthase	2
Chitinase	4	ABC transporter	2
Glucan endo-1,3-glucosidase	4	Alpha carbonic anhydrase	2
12-Oxophytodienoate reductase	3	Leucoanthocyanidin dioxygenase	2
Beta-fructofuranosidase	3	PDR1	2
E3 ubiquitin-protein ligase	3	Phenylalanine ammonia-lyase	2
Proteinase inhibitor	3	Phospho-2-dehydro-3-deoxyheptonate aldolase	2
Ammonium transporter	2	1-Deoxy-D-xylulose-5-phosphate synthase	2
Benzyl alcohol O-benzoyltransferase	2	Methyltransferase	2
Indole-3-acetate O-methyltransferase	2	Proteinase inhibitor	2
Linoleate lipoxygenase	2	Inactive purple acid phosphatase	2
Lipid transfer protein	2	Lipoxygenase	2
Methyltransferase	2	Glutathione S-transferase	2
Reticuline oxidase	2		
Stilbene synthase	2		
Thioredoxin reductase	2		
VQ motif-containing protein	2		
U-box domain-containing protein	2		

## Data Availability

Transcriptome sequences were deposited to the NCBI database, the BioProject ID containing the SRA data is PRJNA667902.

## Supplementary Materials

Supplementary materials can be found at https://www.mdpi.com/1422-0067/22/4/1505/s1.

## Abbreviations

ABA	Abscisic acid
abs.	Absolute
DEG	Differentially expressed gene
ET	Ethylene
FC	Fold change
FDR	False discovery rate
JA	Jasmonic acid
MeJa	Methyl jasmonate
ROS	Reactive oxygen species
SA	Salicylic acid
TF	Transcription factor
