# Supplementary material for: Transcript Dynamics in Wounded and Inoculated Scots Pine
Source: Int J Mol Sci. 2021 Feb 3;22(4):1505. doi: 10.3390/ijms22041505 (PMC7913219; doi:10.3390/ijms22041505)
Supplement: Supplementary file 1 [file ijms-22-01505-s001.zip › Supplementary Table 3.docx]

**Supplementary Table 3**. A summary about the functions of genes that are differentially regulated comparing wounding and inoculation treatments

| **reference transcript ID** | **Blast2GO annotation** | **Short description** |
| --- | --- | --- |
| comp10846_c0_seq1 | U-box domain-containing protein 6-like isoform X1 | functions as E3 ubiquitin ligase [1]. E3 ubiquitin ligases are involved in a variety of proceses, including plant response to abiotic and biotic stress, plant hormone signaling modulation and others [2]. The primary funciton of E3 ubiquitin ligases is protein ubiquitination which influences the faith of the modified portein. Some of the outcomes may be degradation, change in cellular localisation, altered protein-protein interaction. |
| comp1010735_c0_seq1 | 60S ribosomal protein L13a-4-like | structural element of eukaryotic 60S ribosomal subunit [3], involvemed in regulation of translation (if phosphorylated, in human) [4]. |
| comp52169_c0_seq1 | BAHD acyltransferase DCR | Involved in cuticle formation [5,6]. |
| comp52925_c0_seq1 | CASP-like protein 5A2 | The CASP-like protein 5A2 (as a member of Casparian strip membrane proteins (CASP) family) is involved in interactions with peroxidases and cell wall lignification mechanisms [7,8], indicating a role for this gene in plant defense reactions . |
| comp25646_c0_seq1 | CTLH/CRA C-terminal to LisH motif domain containing protein | The mentioned domains are found in the *Arabidopsis* TOPLESS protein which is a transcriptional repressor [9]. These domains are also found in a plant-specific E3 ligase (with the addition of a RING domain) the expression of which was induced by infection in *Lotus japonicus* roots [10]. The information about this group of proteins in plants is scarce, we refrain from speculations. |
| comp53565_c0_seq1 | epidermis-specific secreted glycoprotein EP1-like | It is speculated that in carrot the EP1 expression leads to limitation of water flow through the cell wall [11] thus suggesting that intentional drying of the affected site might be a Scots pine defence strategy. |
| comp58426_c0_seq1 | ER membrane protein complex subunit 4 | In humans ER membrane protein complex subunit 4 may mediate anti-apoptotic activity [12]. The apparent interaction of the ER membrane protein complex with the ER-associated degradation system [13] could indicate the role of this protein and its subunits in regulation of protein homeostasis. ER membrane protein complex is highly conserved among eukaryotes [14]. |
| comp57456_c0_seq1 | heat shock 22 kDa protein, mitochondrial-like isoform X2 | Heat shock proteins typically respond not only to heat but to oxidative stress as well [15–18]. If an oxidative burst is a plant defence strategy, increased expression of heat shoch proteins could be expected. |
| comp54532_c0_seq1 | L-lactate dehydrogenase A | Dolferus et al. [19] demonstrated that in *Arabidopsis* lactate dehydrogenase gene LDH1 expression is influenced by hypoxia, drought, cold and mechanical wounding. This study also demonstrated that *Arabidopsis* plants can secrete lactate efficiently into the medium, saving the cells from the toxic effects of increased lactate concentration. This suggests lactate could be used as a defence metabolite as lactic acid has inhibitory properties on growth of some fungi. It has to be remembered that the pyruvate to lactate reaction is reversible. |
| comp54163_c0_seq3 | L-type lectin-domain containing receptor kinase S.4-like | Involved in *Arabidopsis* resistance responses against fungi and bacteria [20]. |
| comp28540_c0_seq1 | magnesium-chelatase subunit ChlH, chloroplastic | A substantial amount of information is summarized from seven studies in the Uniprot portal regarding this protein [21]. Citing this resource, "Multifunctional protein involved in chlorophyll synthesis, plastid-to-nucleus retrograde signaling and abscisic acid (ABA) perception. In chlorophyll synthesis, catalyzes the insertion of magnesium ion into protoporphyrin IX to yield Mg-protoporphyrin IX. The reaction takes place in two steps, with an ATP-dependent activation followed by an ATP-dependent chelation step. In addition to its function in the Mg-chelatase enzyme, is required for the plastid-to-nucleus retrograde signaling. The plastid-to-nucleus signal plays an important role in the coordinated expression of both nuclear- and chloroplast-localized genes that encode photosynthesis-related proteins. Has a role in mediating ABA signaling in stomatal guard cells and during seed germination. Binds ABA and is a positive regulator of ABA signaling." |
| comp45373_c0_seq2 | ---NA--- |  |
| comp1970482_c0_seq1 | ---NA--- |  |
| comp54684_c1_seq1 | ---NA--- |  |
| comp40935_c0_seq1 | NAC domain-containing protein 86-like | Arabidopsis NAC86 is a transcription factor which, together with NAC45 leads to a controlled autolysis necessary for sieve element differentiation [22]. |
| comp38130_c0_seq1 | PB1 domain-containing protein | PB1 domain is present in auxin response factor (ARF) and auxin/indole acetic acid (Aux/IAA) repressors which are both transcription factors [23]. |
| comp50080_c0_seq8 | polyadenylate-binding protein 2 isoform X1 | important in pre-mRNA processing and translation initiation [24]. |
| comp40240_c0_seq2 | pre-mRNA-splicing factor ISY1 homolog | involved in splicing [25], involved in cell cycle regulation [26]. |
| comp40067_c0_seq1 | protein TIME FOR COFFEE-like isoform X1 | involved in circadian clock mechanisms [27], involved in trascription regulation and jasmonate signaling [28]. |
| comp45446_c0_seq1 | putative DEAD-box ATP-dependent RNA helicase 29 isoform X2 | Overexpression of a DEAD-Box RNA Helicase in *Arabidopsis* has been shown to increase salt tolerance [29]. A review by Liu and Imai [30] summarizes that these RNA helicases are important in ribosome and chloroplast ribosome biogenesis, different types of splicing and other processes, some being important in stress response and ABA and auxin signaling. |
| comp40886_c0_seq1 | RmlC-like cupins superfamily protein | RmlC is necessary for synthesis of l-rhamnose, a component of plant cell wall [31]. Cupins are a diverse group of proteins best known as seed storage proteins but other members include sucrose binding proteins, auxin binding proteins and dioxygenase enzymes, as reviewed by Gábrišová et al. [32]. |
| comp52309_c0_seq4 | serine/threonine protein phosphatase 2A regulatory subunit B beta-like | Important in plant metabolism, development, stress response and signal transduction; positively affects β-oxidation of fatty acids and protoauxins [33]. An example of protoauxin oxidation is the oxidation of indole-3-butyric acid (IBA) to indole-3-acetic acid (IAA), auxin [34]. Catalytic subunit of serine/threonine protein phosphatase 2A is a negative regulator of abscisic acid signalling [35]. |
| comp20379_c0_seq1 | shaggy-related protein kinase epsilon | Arabidopsis shaggy-related protein kinase epsilon (synonym Shaggy-related protein kinase 13) (gene name ASK5, Synonym SK13) isinvolved in brassinosteroid signaling which affects transcription [36]. Shaggy-like kinases are also known as glycogen synthase kinase 3 (GSK3) proteins. |
| comp19180_c0_seq1 | tetraspanin-3-like | As reviewed by Reimann et al. [37], plant tetraspanins are small transmembrane proteins which form tetraspanin-enriched microdomains that function as mobile signaling hubs within membranes. The same review mentions that, in rice, promoter regions of tetraspanin genes contain abscisic acid or methyljasmonate-responsive elements. |
| comp20243_c0_seq2 | tRNA:m(4)X modification enzyme TRM13 homolog isoform X1 | Increased expression of rice TRM13 (OsTRM13) is significant for increased salt tolerance [38]. As reviewed in the mentioned publication, it has been proposed that tRNA nucleoside modification links environmental stimuli to the translation mechanism. |
| comp55480_c0_seq16 | UDP-glycosyltransferase 86A1 | mycotoxin detoxification [39] or defense-related compound modification [40]. Regarding the D group, their induction is methyljasmonate independent, but partially salicylic acid dependent. |
| comp55480_c0_seq6 | UDP-glycosyltransferase 86A1 |  |
| comp42833_c0_seq1 | uncharacterized protein LOC18439758 |  |
| comp50512_c0_seq1 | unknown |  |
| comp54196_c0_seq3 | Vacuolar protein sorting-associated protein 13 domain | In *Saccharomyces cerevisiae* vacuolar protein sorting-associated protein 13 is required for sorting signal-dependent recycling of membrane proteins [41]. Biological process gene ontology descriptions include protein targeting to vacuole, protein retention in Golgi apparatus, regulation of inclusion body assembly, mitochondrion organization an others. |
| comp55005_c0_seq1 | paladin isoform X2 | Insufficient information |
| comp41900_c0_seq2 | chaperone protein dnaJ 10 | Analysis of descriptions of DnaJ protein C/III subfamily members [42] provides a description "Plays a continuous role in plant development probably in the structural organization of compartments" [43]. Besides, the dnaJ49 protein is mentioned to participate in heat shock protein binding, cellular response to misfolded protein and ubuquitin-dependent ER associated degradation pathway [44]. |

Paladin isoform X2 and chaperone protein dnaJ 10 are down-regulated.

1. PUB6 - U-box domain-containing protein 6 - Arabidopsis thaliana (Mouse-ear cress) - PUB6 gene & protein Available online: https://www.uniprot.org/uniprot/O48700 (accessed on Oct 8, 2020).

2. Yee, D.; Goring, D.R. The diversity of plant U-box E3 ubiquitin ligases: From upstream activators to downstream target substrates. In Proceedings of the Journal of Experimental Botany; Oxford Academic, 2009; Vol. 60, pp. 1109–1121.

3. Klinge, S.; Voigts-Hoffmann, F.; Leibundgut, M.; Arpagaus, S.; Ban, N. Crystal structure of the eukaryotic 60S ribosomal subunit in complex with initiation factor 6. *Science (80-. ).* **2011**, *334*, 941–948, doi:10.1126/science.1211204.

4. Mazumder, B.; Sampath, P.; Seshadri, V.; Maitra, R.K.; DiCorleto, P.E.; Fox, P.L. Regulated release of L13a from the 60S ribosomal subunit as a mechanism of transcript-specific translational control. *Cell* **2003**, *115*, 187–198, doi:10.1016/S0092-8674(03)00773-6.

5. Panikashvili, D.; Shi, J.X.; Schreiber, L.; Aharoni, A. The arabidopsis DCR encoding a soluble BAHD acyltransferase is required for cutin polyester formation and seed hydration properties. *Plant Physiol.* **2009**, *151*, 1773–1789, doi:10.1104/pp.109.143388.

6. Domínguez, E.; Heredia-Guerrero, J.A.; Heredia, A. Plant cutin genesis: Unanswered questions. *Trends Plant Sci.* 2015, *20*, 551–558.

7. Roppolo, D.; De Rybel, B.; Tendon, V.D.; Pfister, A.; Alassimone, J.; Vermeer, J.E.M.; Yamazaki, M.; Stierhof, Y.D.; Beeckman, T.; Geldner, N. A novel protein family mediates Casparian strip formation in the endodermis. *Nature* **2011**, *473*, 381–384, doi:10.1038/nature10070.

8. Roppolo, D.; Boeckmann, B.; Pfister, A.; Boutet, E.; Rubio, M.C.; Dénervaud-Tendon, V.; Vermeer, J.E.M.; Gheyselinck, J.; Xenarios, I.; Geldner, N. Functional and evolutionary analysis of the CASPARIAN STRIP MEMBRANE DOMAIN PROTEIN family. *Plant Physiol.* **2014**, *165*, 1709–1722, doi:10.1104/pp.114.239137.

9. Martin-Arevalillo, R.; Nanao, M.H.; Larrieu, A.; Vinos-Poyo, T.; Mast, D.; Galvan-Ampudia, C.; Brunoud, G.; Vernoux, T.; Dumas, R.; Parcy, F. Structure of the Arabidopsis TOPLESS corepressor provides insight into the evolution of transcriptional repression. *Proc. Natl. Acad. Sci. U. S. A.* **2017**, *114*, 8107–8112, doi:10.1073/pnas.1703054114.

10. Yuan, S.; Zhu, H.; Gou, H.; Fu, W.; Liu, L.; Chen, T.; Ke, D.; Kang, H.; Xie, Q.; Hong, Z.; et al. A ubiquitin ligase of symbiosis receptor kinase involved in nodule organogenesis. *Plant Physiol.* **2012**, *160*, 106–117, doi:10.1104/pp.112.199000.

11. Van Engelen, F.A.; Hartog, M. V.; Thomas, T.L.; Taylor, B.; Sturm, A.; Van Kammen, A.; De Vries, S.C. The carrot secreted glycoprotein gene EP1 is expressed in the epidermis and has sequence homology to Brassica S-locus glycoproteins. *Plant J.* **1993**, *4*, 855–862, doi:10.1046/j.1365-313x.1993.04050855.x.

12. EMC4 - ER membrane protein complex subunit 4 - Homo sapiens (Human) - EMC4 gene & protein Available online: https://www.uniprot.org/uniprot/Q5J8M3 (accessed on Oct 8, 2020).

13. Christianson, J.C.; Olzmann, J.A.; Shaler, T.A.; Sowa, M.E.; Bennett, E.J.; Richter, C.M.; Tyler, R.E.; Greenblatt, E.J.; Wade Harper, J.; Kopito, R.R. Defining human ERAD networks through an integrative mapping strategy. *Nat. Cell Biol.* **2012**, *14*, 93–105, doi:10.1038/ncb2383.

14. Wideman, J.G. The ubiquitous and ancient ER membrane protein complex (EMC): Tether or not? *F1000Research* **2015**, *4*, 624, doi:10.12688/f1000research.6944.2.

15. Helm, K.W.; Schmeits, J.; Vierling, E. An endomembrane-localized small heat-shock protein from Arabidopsis thaliana. *Plant Physiol.* **1995**, *107*, 287–288, doi:10.1104/pp.107.1.287.

16. Heckathorn, S.A.; Downs, C.A.; Coleman, J.S. Small heat shock proteins protect electron transport in chloroplasts and mitochondria during stress. *Am. Zool.* **1999**, *39*, 865–876, doi:10.1093/icb/39.6.865.

17. Scarpeci, T.E.; Zanor, M.I.; Valle, E.M. Investigating the role of plant heat shock proteins during oxidative stress. *Plant Signal. Behav.* **2008**, *3*, 856–857, doi:10.4161/psb.3.10.6021.

18. Avelange-Macherel, M.-H.; Rolland, A.; Hinault, M.-P.; Tolleter, D.; Macherel, D. The Mitochondrial Small Heat Shock Protein HSP22 from Pea is a Thermosoluble Chaperone Prone to Co-Precipitate with Unfolding Client Proteins. *Int. J. Mol. Sci.* **2020**, *21*, 97, doi:10.3390/ijms21010097.

19. Dolferus, R.; Wolansky, M.; Carroll, R.; Miyashita, Y.; Ismond, K.; Good, A. Functional analysis of lactate dehydrogenase during hypoxic stress in Arabidopsis. *Funct. Plant Biol.* **2008**, *35*, 131, doi:10.1071/FP07228.

20. Wang, Y.; Bouwmeester, K.; Beseh, P.; Shan, W.; Govers, F. Phenotypic analyses of Arabidopsis T-DNA insertion lines and expression profiling reveal that multiple L-type lectin receptor kinases are involved in plant immunity. *Mol. Plant-Microbe Interact.* **2014**, *27*, 1390–1402, doi:10.1094/MPMI-06-14-0191-R.

21. CHLH - Magnesium-chelatase subunit ChlH, chloroplastic precursor - Arabidopsis thaliana (Mouse-ear cress) - CHLH gene & protein Available online: https://www.uniprot.org/uniprot/Q9FNB0 (accessed on Oct 8, 2020).

22. Furuta, K.M.; Yadav, S.R.; Lehesranta, S.; Belevich, I.; Miyashima, S.; Heo, J. ok; Vatén, A.; Lindgren, O.; De Rybel, B.; Van Isterdael, G.; et al. Plant development. Arabidopsis NAC45/86 direct sieve element morphogenesis culminating in enucleation. *Science* **2014**, *345*, 933–937, doi:10.1126/science.1253736.

23. Korasick, D.A.; Westfall, C.S.; Lee, S.G.; Nanao, M.H.; Dumas, R.; Hagen, G.; Guilfoyle, T.J.; Jez, J.M.; Strader, L.C. Molecular basis for AUXIN RESPONSE FACTOR protein interaction and the control of auxin response repression. *Proc. Natl. Acad. Sci. U. S. A.* **2014**, *111*, 5427–5432, doi:10.1073/pnas.1400074111.

24. Palanivelu, R.; Belostotsky, D.A.; Meagher, R.B. Arabidopsis thaliana poly (A) binding protein 2 (PAB2) functions in yeast translational and mRNA decay processes. *Plant J.* **2000**, *22*, 187–198, doi:10.1046/j.1365-313X.2000.00721.x.

25. Dix, I.; Russell, C.; Yehuda, S. Ben; Kupiec, M.; Beggs, J.D. The identification and characterization of a novel splicing protein, Isy1p, of Saccharomyces cerevisiae. *RNA* **1999**, *5*, 360–368, doi:10.1017/S1355838299981396.

26. Dahan, O.; Kupiec, M. Mutations in genes of Saccharomyces cerevisiae encoding pre-mRNA splicing factors cause cell cycle arrest through activation of the spindle checkpoint. *Nucleic Acids Res.* **2002**, *30*, 4361–4370, doi:10.1093/nar/gkf563.

27. Hall, A.; Bastow, R.M.; Davis, S.J.; Hanano, S.; McWatters, H.G.; Hibberd, V.; Doyle, M.R.; Sung, S.; Halliday, K.J.; Amasino, R.M.; et al. The Time for Coffee Gene Maintains the Amplitude and Timing of Arabidopsis Circadian Clocks. *Plant Cell* **2003**, *15*, 2719–2729, doi:10.1105/tpc.013730.

28. Shin, J.; Heidrich, K.; Sanchez-Villarreal, A.; Parker, J.E.; Davis, S.J. TIME FOR COFFEE represses accumulation of the MYC2 transcription factor to provide time-of-day regulation of jasmonate signaling in Arabidopsis. *Plant Cell* **2012**, *24*, 2470–2482, doi:10.1105/tpc.111.095430.

29. Nguyen, L.; Seok, H.-Y.; Woo, D.-H.; Lee, S.-Y.; Moon, Y.-H. Overexpression of the DEAD-Box RNA Helicase Gene AtRH17 Confers Tolerance to Salt Stress in Arabidopsis. *Int. J. Mol. Sci.* **2018**, *19*, 3777, doi:10.3390/ijms19123777.

30. Liu, Y.; Imai, R. Function of Plant DExD/H-Box RNA Helicases Associated with Ribosomal RNA Biogenesis. *Front. Plant Sci.* **2018**, *9*, 125, doi:10.3389/fpls.2018.00125.

31. Watt, G.; Leoff, C.; Harper, A.D.; Bar-Peled, M. A bifunctional 3,5-epimerase/4-keto reductase for nucleotide-rhamnose synthesis in arabidopsis. *Plant Physiol.* **2004**, *134*, 1337–1346, doi:10.1104/pp.103.037192.

32. Gábrišová, D.; Klubicová, K.; Danchenko, M.; Gömöry, D.; Berezhna, V. V.; Skultety, L.; Miernyk, J.A.; Rashydov, N.; Hajduch, M. Do Cupins Have a Function Beyond Being Seed Storage Proteins? *Front. Plant Sci.* **2016**, *6*, 1215, doi:10.3389/fpls.2015.01215.

33. Kataya, A.R.A.; Heidari, B.; Hagen, L.; Kommedal, R.; Slupphaug, G.; Lillo, C. Protein phosphatase 2A holoenzyme is targeted to peroxisomes by piggybacking and positively affects peroxisomal b-oxidation. *Plant Physiol.* **2015**, *167*, 493–506, doi:10.1104/pp.114.254409.

34. Strader, L.C.; Wheeler, D.L.; Christensen, S.E.; Berens, J.C.; Cohen, J.D.; Rampey, R.A.; Bartel, B. Multiple facets of Arabidopsis seedling development require indole-3-butyric acid-derived auxin. *Plant Cell* **2011**, *23*, 984–999, doi:10.1105/tpc.111.083071.

35. Pernas, M.; García-Casado, G.; Rojo, E.; Solano, R.; Sánchez-Serrano, J.J. A protein phosphatase 2A catalytic subunit is a negative regulator of abscisic acid signalling1. *Plant J.* **2007**, *51*, 763–778, doi:10.1111/j.1365-313X.2007.03179.x.

36. Zhu, J.Y.; Li, Y.; Cao, D.M.; Yang, H.; Oh, E.; Bi, Y.; Zhu, S.; Wang, Z.Y. The F-box Protein KIB1 Mediates Brassinosteroid-Induced Inactivation and Degradation of GSK3-like Kinases in Arabidopsis. *Mol. Cell* **2017**, *66*, 648-657.e4, doi:10.1016/j.molcel.2017.05.012.

37. Reimann, R.; Kost, B.; Dettmer, J. TETRASPANINs in plants. *Front. Plant Sci.* 2017, *8*, 545.

38. Wang, Y.; Li, D.; Gao, J.; Li, X.; Zhang, R.; Jin, X.; Hu, Z.; Zheng, B.; Persson, S.; Chen, P. The 2′-O-methyladenosine nucleoside modification gene OsTRM13 positively regulates salt stress tolerance in rice. *J. Exp. Bot.* **2017**, *68*, 1479–1491, doi:10.1093/jxb/erx061.

39. He, Y.; Ahmad, D.; Zhang, X.; Zhang, Y.; Wu, L.; Jiang, P.; Ma, H. Genome-wide analysis of family-1 UDP glycosyltransferases (UGT) and identification of UGT genes for FHB resistance in wheat (Triticum aestivum L.). *BMC Plant Biol.* **2018**, *18*, doi:10.1186/s12870-018-1286-5.

40. Li, Y.; Baldauf, S.; Lim, E.-K.; Bowles, D.J. Phylogenetic Analysis of the UDP-glycosyltransferase Multigene Family of Arabidopsis thaliana* □ S. **2000**, doi:10.1074/jbc.M007447200.

41. VPS13 - Vacuolar protein sorting-associated protein 13 - Saccharomyces cerevisiae (strain ATCC 204508 / S288c) (Baker’s yeast) - VPS13 gene & protein Available online: https://www.uniprot.org/uniprot/Q07878 (accessed on Oct 9, 2020).

42. family:"dnaj family c iii subfamily" in UniProtKB Available online: https://www.uniprot.org/uniprot/?query=family:%22DnaJ+family.+C%2FIII+subfamily%22&sort=score (accessed on Dec 29, 2020).

43. ATJ10 - Chaperone protein dnaJ 10 - Arabidopsis thaliana (Mouse-ear cress) - ATJ10 gene & protein Available online: https://www.uniprot.org/uniprot/Q8GYX8 (accessed on Dec 29, 2020).

44. ATJ49 - Chaperone protein dnaJ 49 - Arabidopsis thaliana (Mouse-ear cress) - ATJ49 gene & protein Available online: https://www.uniprot.org/uniprot/Q9FH28 (accessed on Dec 29, 2020).
