# Supplementary figures and images for: Transcript Dynamics in Wounded and Inoculated Scots Pine
Source: Int J Mol Sci. 2021 Feb 3;22(4):1505. doi: 10.3390/ijms22041505 (PMC7913219; doi:10.3390/ijms22041505)

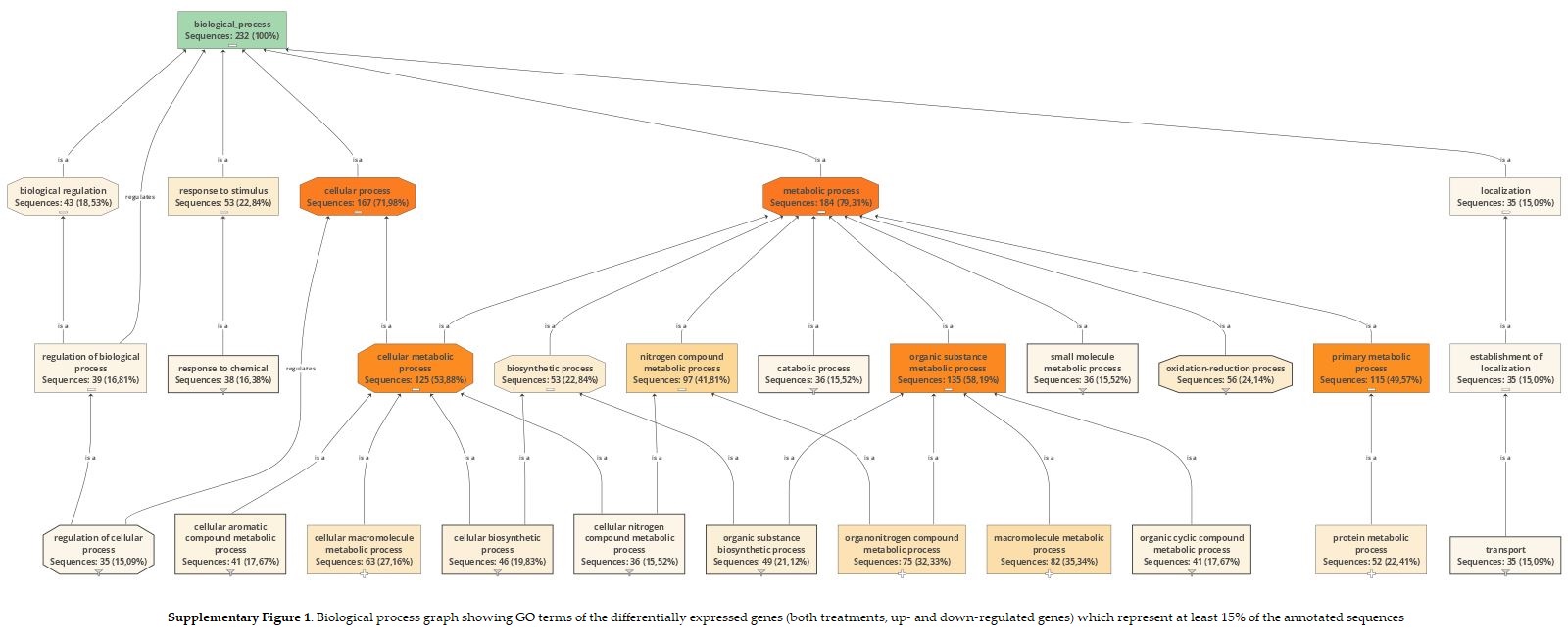

Supplement: Supplementary file 1 [file ijms-22-01505-s001.zip › Supplementary Figure 1 with caption.JPG]

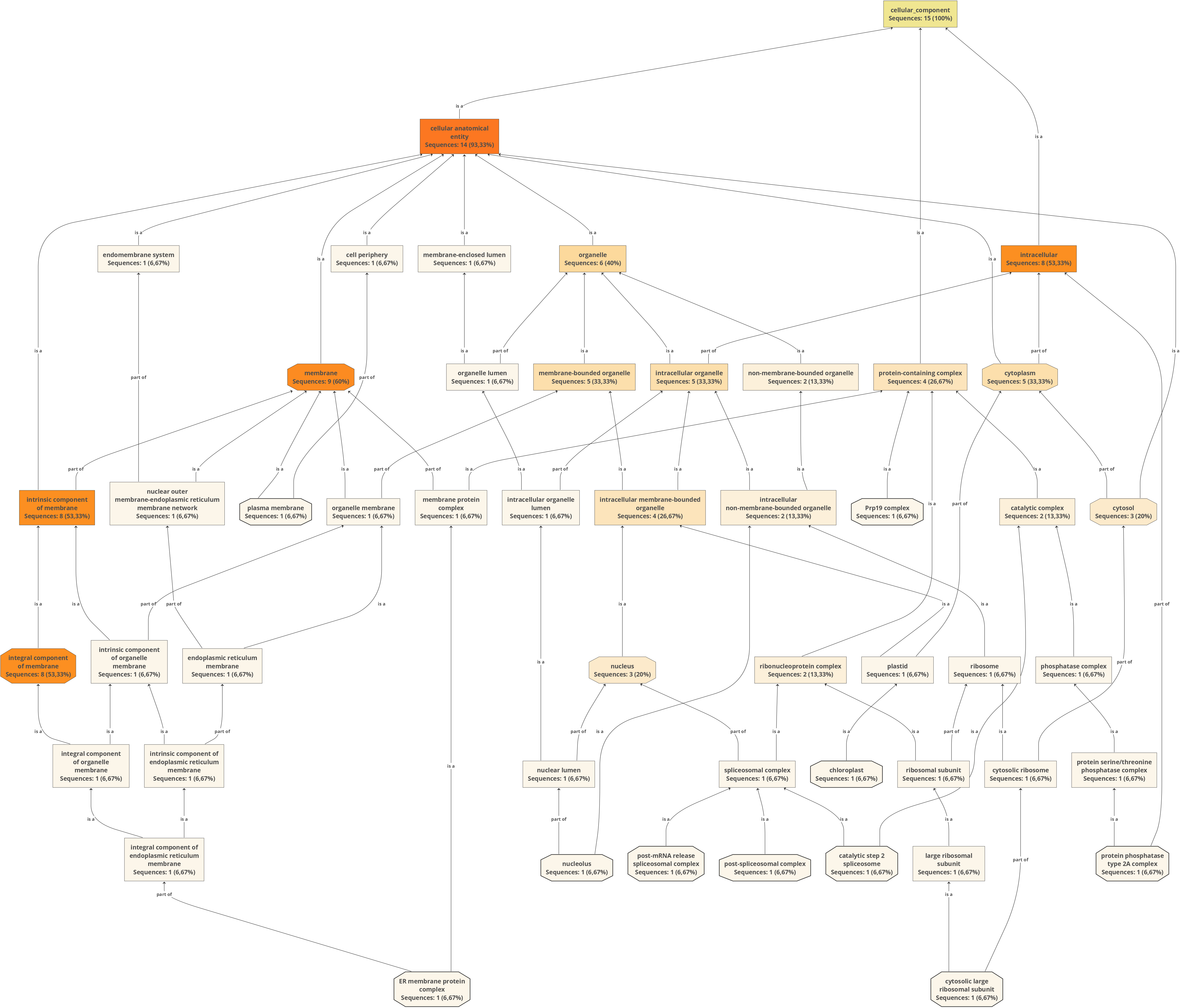

Supplement: Supplementary file 1 [file ijms-22-01505-s001.zip › Supplementary Figure 2 Cellular localization gene ontology graph - with caption.png]
